# COVID-19 case findings and contact tracing in South German nursing homes

**DOI:** 10.1186/s12879-022-07133-8

**Published:** 2022-03-29

**Authors:** Linda Eichner, Christiane Schlegel, Gottfried Roller, Hannes Fischer, Rieke Gerdes, Felix Sauerbrey, Sarah Schönleber, Franziska Weinhart, Martin Eichner

**Affiliations:** 1 Public Health Department Reutlingen, St. Wolfgangstr. 13, 72764 Reutlingen, Germany; 2 Medical Faculty, University of Tübingen, Tübingen, Germany; 3 Epimos GmbH, Bischofsheim in der Rhön, Germany; 4 Institute for Clinical Epidemiology and Applied Biometry, Tübingen, Germany

**Keywords:** Outbreak management, Testing strategy, COVID-19, Nursing homes, Asymptomatic cases

## Abstract

**Background:**

The air borne SARS-CoV-2 poses a high threat to the elderly and people with underlying diseases. COVID-19 spread quickly in South German nursing homes and for this reason called for preventive measures by the German government. The aim of this paper is to showcase the testing strategies implemented by the Public Health Department Reutlingen to control the spread of COVID-19 in local nursing homes and to report the results thereof.

**Methods:**

This study reports COVID-19 outbreaks in nursing homes in Reutlingen County and how they were dealt with through extensive testing, contact tracing, isolation and hygiene inspections. The testing strategy consisted of three phases: In phase 1 only suspected cases, in phase 2 all staff and residents, and in phase 3 all suspected cases and their contacts were tested.

**Results:**

Nearly all residents (98%) and staff members (92%) of all nursing homes in Reutlingen County were tested for SARS-COV-2. 25 of 37 nursing homes had COVID-19 cases, 5 had 30–81 cases/home. 62% of the 395 nursing homes cases were residents, but less than half of them exhibited symptoms (41%). The cases uncovered in nursing homes represented 26% of all 1529 cases in Reutlingen County during the time of this study.

**Conclusions:**

Many COVID-19 cases were discovered through extensive testing, allowing for early interventions. The results shed light on the COVID-19 situation in nursing homes and allowed for individually designed preventive measures. The results also lead to a change in the German legislation. The outbreak management methods of the Public Health Department Reutlingen may also be applicable in other countries.

**Supplementary Information:**

The online version contains supplementary material available at 10.1186/s12879-022-07133-8.

## Background

Since the end of 2019, COVID-19 (Coronavirus Disease 2019), an airborne infectious disease caused by SARS-CoV-2 (severe acute respiratory syndrome virus 2), has been sweeping the globe in a world-wide pandemic [1]. SARS-CoV-2 is known to lead to a more severe course of infection for certain high risk groups, particularly the elderly in nursing homes. This poses a monumental challenge for countries with a very old population [2]. Germany, one of the top countries with the most COVID-19 cases in Europe (10,670,322 cases as of 4 February, 2022), incidentally also has one of the oldest populations in the EU: 28.5% are ≥ 60 years old [3, 4].

**Fig. 1:**
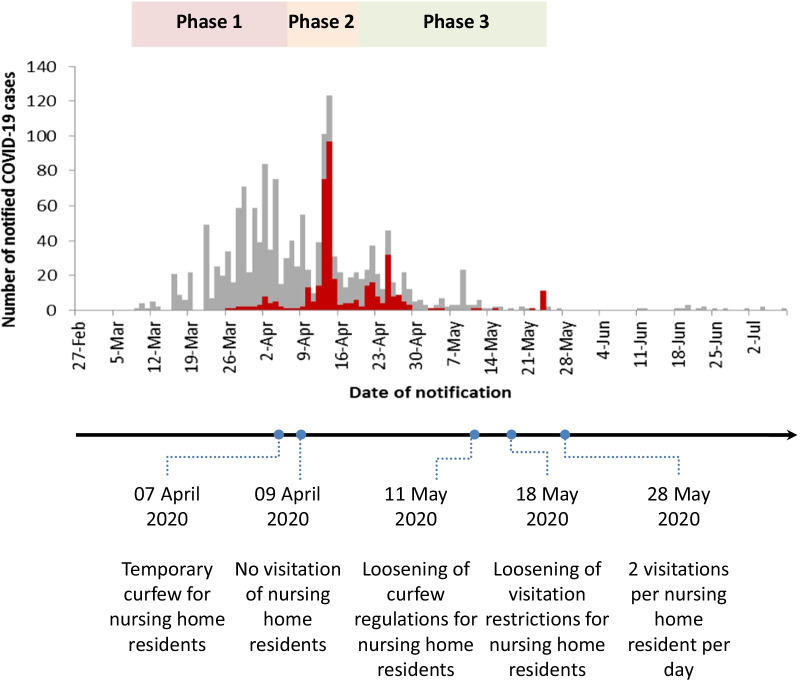
COVID-19 cases in nursing homes (red) and the general population (grey). Underneath the graph, the legal ordinances regarding COVID-19 in nursing homes that were passed in BW are shown [4–8]. Most nursing home cases occurred relatively late compared to the ongoing pandemic in the general population

After the spring holidays of 2020, vacationers returned from high risk countries and brought the virus with them. Shortly after the first COVID-19 cases appeared in Baden-Württemberg (BW), South Germany, that nursing homes reported their first cases. In Reutlingen County, BW, only 2 weeks after the first COVID-19 case had been discovered, the first nursing homes were known to be affected. During the beginning of the pandemic, the German government issued several COVID-19 related ordinances and recommendations for nursing homes to protect the most vulnerable groups of our society (Fig. 1) [5–9]. By banning practically all visitations to nursing homes and by forbidding residents to leave (unless urgently required), the government tried to form protective bubbles around these facilities [5, 6]. As the public health system was quickly over-burdened by COVID-19 cases, the Public Health Department Reutlingen (PHDR) had to act fast and take over the testing of suspected cases. Personal protective equipment (PPE), disinfectants and testing equipment were sparse in the early phase of the pandemic, making it nearly impossible for general practitioners to cope with the rising demand [10]. Within few days, the PHDR provided two drive-in centres, a mobile testing station and a call centre. However, the PHDR also put a strong emphasis on the local nursing homes that were heavily affected. Over an extended time period, the PHDR went through different testing strategies in combination with intensified hygienic measures to prevent the spread of COVID-19 in local nursing homes (see Fig. 1). The aim of this paper is to showcase the methods implemented by the PHDR to control the spread of COVID-19 in local nursing homes and to report the results thereof.

## Methods

### Study population

Reutlingen County is a mainly rural county located in the Federal State of BW, South Germany, and has 286,748 inhabitants; 1985 of these inhabitants are living in one of the 37 nursing homes and are looked after by 2510 staff members. One of the nursing homes caters towards residents of varying ages with neurological diseases. 27% of the residents and 17% of the staff members are male. The ages of residents ranged from 26 to 106 years (median 86 years) and that of staff members (including volunteer workers) ranged from 16 to 84 years (median 45 years). During the time of this study the nursing homes did not accept new residents.

### Test strategies

Swab samples were taken in the nursing homes by physicians employed by the PHDR. Laboratory sequencing of the swabs was conducted via polymerase chain reaction. The related costs for the swab tests were covered by the German government and the health insurances of the tested individuals. All positive SARS-COV-2 test results in Reutlingen County had to be reported to the PHDR according §7 to the German National Infection Protection Law—IfSG [11].

### Phase 1: Testing of suspected cases (March 8–April 5, 2020)

Since it is known that elderly people have a particularly high risk of suffering a severe infection, the PHDR decided to extend their testing towards nursing home residents and staff members [2]. From March 8 until April 5, 2020, nursing home residents and nursing staff members with acute respiratory symptoms or fever or pneumonia were tested for SARS-CoV-2.

### Phase 2: Screenings (April 6–20, 2020)

Studies indicate that about 56% of potentially contagious SARS-COV-2 geriatric cases are asymptomatic and pre-symptomatic, indicating that only testing symptomatic people (as recommended at that time) would miss many cases of those who could infect others [12, 13]. With COVID-19 numbers continually rising in the population and nursing homes, the PHDR made the drastic decision to screen all staff members and residents of all nursing homes in Reutlingen County to break the COVID-19 wave.

From April 6–20, 2020, all residents and staff members of all 37 nursing homes in Reutlingen County were offered a SARS-CoV-2 swab test through mobile testing services. Non-nursing staff members and volunteer workers were also included in this screening (together with nursing staff members, they are classified as “staff” in this paper).

### Phase 3: Contact tracing (April 21–May 23, 2020)

From 21 April until 23 May, symptomatic residents and staff members without a positive test result were classified as suspected cases. In “hot spot nursing homes” where ≥ 40 confirmed cases had occurred, all residents and staff members who did not yet have a positive test result were tested as soon as another person developed general COVID-19 related symptoms. In homes with < 40 cases, merely the immediate contacts of symptomatic suspected cases were tested. Residents and staff members that had not yet participated in the screening were also eligible to get tested if required.

### Collection of personal data

The PHDR collected personal data of the tested people regarding age, gender, symptoms, and name of facility—in accordance with the German National Infection Protection Act—IfSG [11]. For people with positive SARS-CoV-2 results, pre-existing illnesses were also recorded, and the clinical course of the infection was monitored daily as a medical journal for 2 weeks after the test results were notified.

### Quarantine

Residents who were tested positive and those who were tested negative for SARS-CoV-2 were isolated in separate groups. Staff members with a positive test result had to self-isolate at home. Isolation usually ended 2 weeks after the test date. However, in facilities with ≥ 30 confirmed cases isolation ended after 3 weeks. All residents and staff members with a negative test result were monitored daily for symptoms for 2 weeks. If they subsequently developed COVID-19 related symptoms, they and their contacts were immediately reported to the PHDR and tested for SARS-CoV-2. If any person tested positive, he or she was isolated as described above.

### Monitoring and support through the PHDR

During all phases, the PHDR kept close contact with the nursing homes, monitored the progression of their outbreaks, offering consultations and ordained hygienic and preventative measures. All COVID-19 patients were monitored daily and the PHDR was notified when symptoms worsened, when patients were transferred to hospital or passed away. The hygiene and quarantine measures of facilities with ≥ 10 confirmed cases were inspected at least once by the PHDR. Facilities with ≥ 40 cases were inspected weekly until the hygienic standards were deemed satisfactory by the PHDR.

During the COVID-19 pandemic, all facility staff members had to wear full PPE. Pandemic officers were appointed in all facilities (including homes without cases). They implemented and monitored hygiene standards and procedures. Residents were not allowed to leave the nursing homes or to have visitors, unless there was an emergency, according to German laws at that time [6]. Exceptional visitors had to wear full PPE.

The SARS-CoV-2 positive and negative cohorts were looked after by staff members who had a negative test result. There was no exchange of nursing staff between these cohort groups. Only if there was a severe shortage in nursing staff, asymptomatic staff members with a positive test result were allowed to care for the positive cohort’s residents, avoiding all contact with people who were not tested positive for SARS-CoV-2. The PHDR established contacts between the nursing homes and the Red Cross to provide additional nursing staff members during immediate shortages, and with the Administrative District Office of Reutlingen for the provision of additional PPE and disinfectants if required.

## Results

**Fig. 2:**
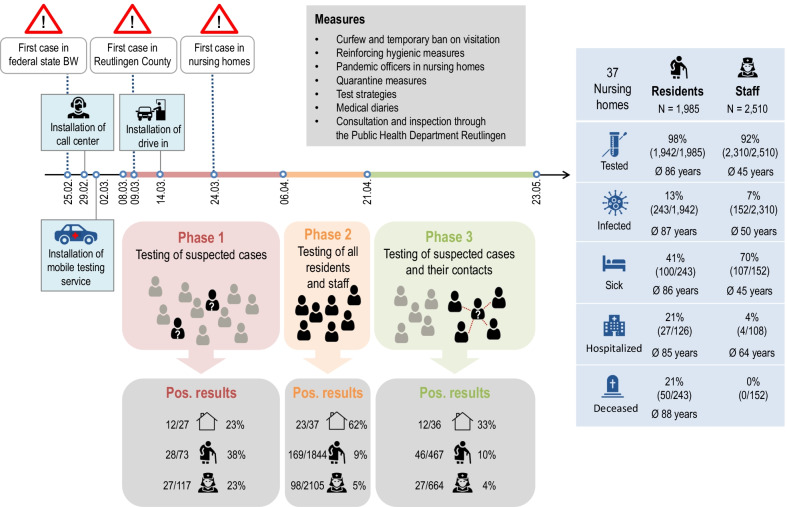
Outbreak management and test strategies of the PHDR in nursing homes in Reutlingen County. Icons of Freepik, fjstudio, Smashpik and Pixel perfect (www.flaticon.com) modified by the authors

On March 24, 2020, about 2 weeks after the first COVID-19 case had been discovered in Reutlingen County, the first COVID-19 cases appeared in nursing home staff and residents. Within few days, 12 of the 37 nursing homes reported COVID-19 cases; several of them had clusters, ranging from 2–18 cases. 28/73 (38%) tested nursing home residents and 27/117 (23%) tested nursing home employees with symptoms were positive for SARS-CoV-2, accounting for 8% of all 681 confirmed cases in the county by April 5 2020 (Fig. 2).

During the screening (April 6–20, 2020), 92.9% of all 1985 residents and 83.9% of all 2510 staff members were tested for SARS-CoV-2. In 23/37 nursing homes, 267 new COVID-19 cases were detected; 63% of them were residents (Fig. 2). 94% of the cases found were in 12 nursing homes with ongoing COVID-19 outbreaks.

Merely a day after the screening of all nursing homes had been completed; the first residents and staff members of the COVID-19 negative cohorts developed symptoms and triggered a new wave of testing. A total of 86 suspected cases lead up to the examination of 1045 asymptomatic contacts (440 residents and 605 staff members; Additional file 1: S4). Only 19/86 suspected cases were positive for SARS-CoV-2, but the examination of their contacts led to the discovery of 54 additional COVID-19 cases. 63% of the 73 new cases were residents (Fig. 2). Most nursing homes with 1–2 cases in phases 1 and 2 did not have more than 1–4 new cases in phase 3.

**Fig. 3:**
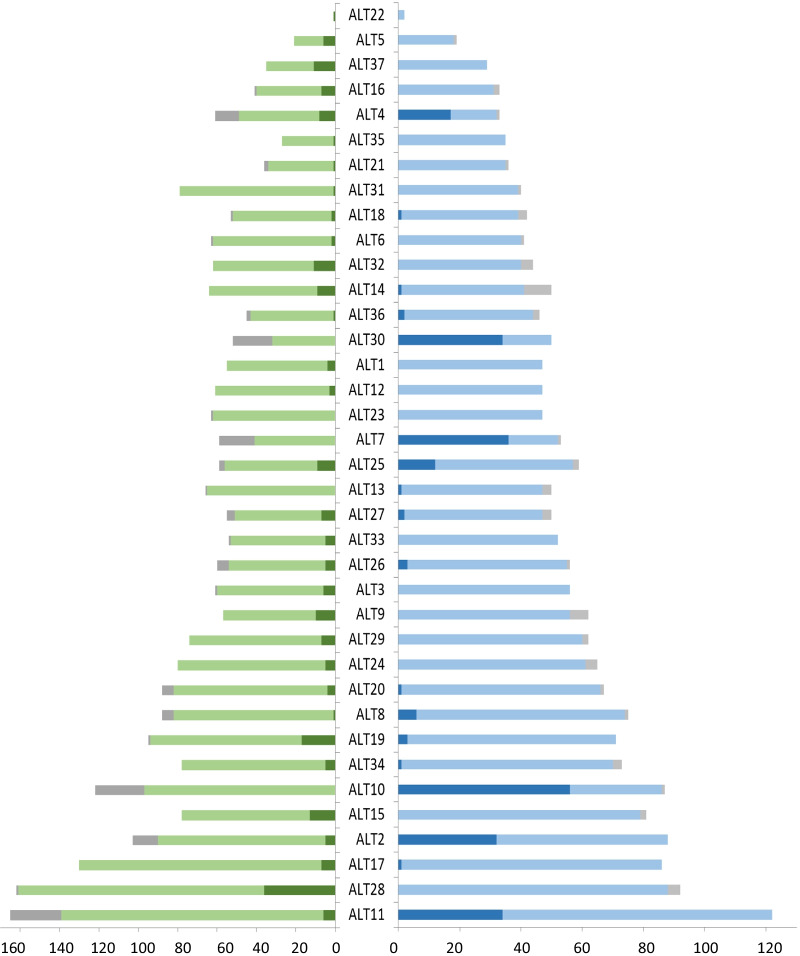
Test results for staff members (left) and residents (right) per nursing home (ALT). Dark green: staff members with a positive test result; green: staff members with only negative test results; dark grey: staff members without a test; dark blue: residents with a positive test result; blue: residents with only negative test results; light grey: residents without a test

By May 23rd 2020, 5319 SARS-CoV-2 tests had been conducted in the nursing homes, which accounted for about a third of all tests conducted by the PHDR in that period. 98% of all 1985 residents and 92% of all 2510 nursing home staff members were tested at least once for SARS-CoV-2, leading to the discovery of 395 COVID-19 cases in 25/37 nursing homes (26% of all 1529 cases in Reutlingen County); 5 nursing homes had 31–81 cases/home (Fig. 3, Additional file 1: S7). In four nursing homes over 50% of their residents tested positive for SARS-COV-2 (53–77%).

Most cases were residents (62%) with a median age of 86 years (range 53–106). The infected staff members had a median age of 45 years (range 17–82). Only 41% of 243 infected residents exhibited symptoms, whereas 71% of 152 infected staff members had symptoms (Fig. 3). In phase 2, 21% of the 63 infected residents with a medical journal only developed symptoms after the test was conducted (Additional file 1: S3).

Many infected residents suffered from psychological (54.2%) or neurological disorders (50.0%). The most common underlying diseases among residents with COVID-19 (243) were heart diseases (44%), retinal diseases (23%), and type II diabetes (22%; Additional file 1: S5). Subliminal symptoms like joint pain, head ache and sore throat were reported more frequently from staff members than from residents (39.7% and 9.0%, respectively), yet fever and pneumonia were more frequent among residents than among staff members (33.3% and 3.4%, respectively; Additional file 1: S3). A total of 27 infected residents and 4 infected staff members were hospitalized, and 50 COVID-19 related deaths (out of a total of 83 COVID-19 deaths in Reutlingen County) were reported for nursing home residents (Fig. 2, Table 1, and Additional file 1: S6–S7). Men were more likely than women to get infected, to be admitted to hospital with COVID-19 and to die from COVID-19 (Additional file 1: S1).

**Table 1:** Number of COVID-19 cases, deaths and hospitalizations in nursing homes with > 10 cases

Nursing home	Total no. of COVID-19 cases	No. of residents with COVID-19	No. of staff members with COVID-19	No. of hospitalized COVID-19 cases	No. of COVID-19 related deaths
ALT10	81	56/78 (71%)	25/117 (21%)	3/81 (4%)	17/81 (21%)
ALT11	60	34/116 (29%)	26/150 (17%)	5/60 (8%)	8/60 (13%)
ALT7	54	36/47 (77%)	18/57 (32%)	7/54 (13%)	11/54 (11%)
ALT30	54	34/46 (74%)	20/47 (43%)	5/54 (9%)	3/54 (5%)
ALT2	45	32/80 (40%)	13/95 (14%)	3/45 (7%)	7/45 (15%)
ALT4	29	17/32 (53%)	12/53 (23%)	2/29 (7%)	0/29 (0%)
ALT25	15	12/45 (26%)	3/48 (6%)	1/15 (7%)	0/15 (0%)
ALT8	12	6/69 (9%)	6/84 (7%)	0/12 (0%)	1/12 (8%)

## Discussion

Through extensive testing, the PHDR uncovered many new SARS-CoV-2 infections in two thirds of the nursing homes of Reutlingen County. This provided a better overview of the COVID-19 outbreak situation in local nursing homes and allowed for individually designed preventive measures for each affected institution.

More than half of the infected residents were asymptomatic and most likely contributed to the fast spread in nursing homes. National recommended testing strategies at that time that solely focused on symptomatic cases were probably not sufficient to prevent the spread of infections in nursing homes [14]. The PHDR pioneered in extending testing strategies in nursing homes and thereby potentially prevented further outbreaks. The detection of asymptomatic cases particularly in phases 2 and 3 possibly helped prevent further cases, hospitalizations and deaths. The results of the screenings of nursing homes in Reutlingen County most likely contributed to the decision of the Federal State of BW to recommend screening of all nursing homes (issued on April 29, 2020) [15]. On June 8th, 2020, the national German government also decided that asymptomatic contacts of confirmed cases would also be eligible for tests [16]. Furthermore, the national government expanded their testing strategies in nursing homes with COVID-19 cases, making swab tests available for all staff members and residents of afflicted homes [16]. As the numbers of COVID-19 cases were rising again, the German Government called for broad testing in nursing homes and issued hygienic guidelines for nursing homes on how to handle potential cases [17, 18].

## Conclusion

During a period in which COVID-19 cases were very frequent in the general population, the early testing of asymptomatic nursing home residents and staff members may have played a vital role in preventing new COVID-19 cases. A heightened awareness of the nursing homes in regard to infection transmission, accompanied by the surveillance and support of the local Public Health Departments, played a key role in the prevention of further outbreaks. The control strategies outlined in this paper may also be considered in other countries.

## Supplementary Information

**Additional file 1:** Supplementary Material for Covid-19 finding and contact tracing in South German nursing homes. Detailed results of tested nursing home staff and residents in each testing phase, including number of cases found in each nursing home, as well as gender, underlying medical conditions, symptoms, hospitalizations, and deaths of Covid-19 cases.

## Data Availability

The datasets used and/or analysed during the current study are available from the corresponding author on reasonable request.

## Abbreviations

BW: Baden-Wuerttemberg
COVID-19: Coronavirus Disease 2019
IfSG: German National Infection Control Act
PHDR: Public Health Department Reutlingen
PPE: Personal protective equipment
SARS-CoV-2: Severe acute respiratory syndrome coronavirus 2
