# Supplementary material for: COVID-19 case findings and contact tracing in South German nursing homes
Source: BMC Infect Dis. 2022 Mar 29;22:307. doi: 10.1186/s12879-022-07133-8 (PMC8961262; doi:10.1186/s12879-022-07133-8)
Supplement: Supplementary file 1 — Additional file 1: Supplementary Material for Covid-19 finding and contact tracing in South German nursing homes. Detailed results of tested nursing home staff and residents in each testing phase, including number of cases found in each nursing home, as well as gender, underlying medical conditions, symptoms, hospitalizations, and deaths of Covid-19 cases. [file 12879_2022_7133_MOESM1_ESM.docx]

**Additional file**

**Table of contents**

S1 Gender of SARS-COV-2 cases P. 1

S2 Number of COVID-19 cases in nursing homes with 10 or more cases P. 2

S3 Symptoms P. 3

S4 Testing of suspected symptomatic cases and their contacts P. 4

S5 Underlying medical conditions P. 4

S6 Hospitalizations P. 5

S7 Deaths P. 6

S8 Number of examined and infected staff members and nursing home residents P. 8

**S1 Gender of SARS-COV-2 cases**

Table S1. Genders of infected, hospitalized and deceased COVID-19 cases in nursing homes (residents and staff members).

|  | Female | Male | Total |
| --- | --- | --- | --- |
| Tested | 3293 (78%) | 905 (21%) | 4198* |
| Infected | 302 (76%) | 93 (24%) | 395 |
| Hospitalized | 15 (47%) | 17 (53%) | 32 |
| Deceased | 29 (58%) | 21 (42%) | 50 |

*The gender of additional 45 tested individuals was unknown.

**S2 Number of COVID-19 cases in nursing homes with 10 or more cases**

Figure S1. Cumulative numbers of COVID-19 cases over time in nursing homes with 10 or more cases. Infected nursing home residents are depicted in red and infected staff members in black.

**S3 Symptoms**

Medical diaries were collected from 96.3% out of 243 infected residents and from 96.7% out of 152 infected staff members. Medical diaries with only singular events of nausea, diarrhoea or vomiting and no other symptoms were exempted from this analysis, as it is believed that these symptoms are due to another cause than COVID-19. The frequencies of symptoms obtained from the medical diaries are depicted in Table 2.

Table S2. Symptoms among SARS-CoV-2 positive residents (n = 234) and staff members (n = 147)

| Symptoms | No. of residents | No. of staff members |
| --- | --- | --- |
| Cough | 82 (35.0%) | 79 (53.7%) |
| Fever | 93 (39.7%) | 46 (33.3 %) |
| Running nose | 25 (10.7%) | 58 (39.4 %) |
| Joint pain | 7 (3.0%) | 32 (21.8 %) |
| Sore throat | 6 (2.6%) | 45 (30.6 %) |
| Headache | 2 (0.9 %) | 53 (36.1 %) |
| Nausea | 14 (6.0 %) | 17 (11.7 %) |
| Vomiting | 9 (3.7%) | 4 (2.7 %) |
| Diarrhoea | 19 (8.1 %) | 12 (8.1 %) |
| Shortness of breath | 40 (17.1 %) | 23 (15.6 %) |
| Lung infection | 21 (9.0 %) | 5 (3.4 %) |

During phase 2, it was analysed whether residents or staff members were symptomatic before, during or after the test. 87% of all infected residents (169) and 86% of all infected staff members (98) handed in medical diaries during this phase. Unfortunately, the symptomatic patient’s status before or during the test were not recorded on the 6^th^ and the 7^th^ of April, 2020; thus, these patients were exempted from the following analysis. SARS-CoV-2 cases without a medical diary were also exempted from this analysis. Symptoms in the first 14 days before the swab test, during the swab test and up to 7 days after the swab test were monitored and included in Table 3.

Table S3. Symptoms of test negative and test positive residents and staff members before, during and after the swab test, respectively (8^th^ of April until 20^th^ of April 2020).

|  | sympt.  only before  test | sympt.  only at test | sympt.  only after test | sympt.  before and at test | sympt.  at and after test | sympt.  before and after test | sympt.  before, at and after test | ever sympt. | never sympt. | total sympt. |
| --- | --- | --- | --- | --- | --- | --- | --- | --- | --- | --- |
| Residents  tested positive | 2 | 0 | 13 | 14 | 0 | 7 | 2 | 38 | 25 | 63 |
| Residents  tested  negative | 44 | 23 | 4 | 18 | 0 | 4 | 0 | 93 | 1,503 | 1,596 |
| Staff members  tested positive | 6 | 5 | 0 | 33 | 0 | 4 | 1 | 49 | 14 | 63 |
| Staff members  tested negative | 65 | 64 | 1 | 44 | 0 | 6 | 2 | 182 | 1,352 | 1,534 |

**S4 Testing of suspected symptomatic cases and their contacts**

Whenever a resident or staff member of the negative cohort exhibited general symptoms in phase 2 (April 5^th^ - April 20^th^, 2020), then that person and all of his or her close contacts (classified as having had at least 15 min of face-to-face contact) were tested again for SARS-COV-2. In some cases, this included all people living with the resident. In five nursing homes with 30 or more confirmed cases, the entire negative cohort including staff members and residents were tested again for SARS-COV-2. This testing strategy led to the identification of 73 new cases (see Table 4).

Table S4. Results of case finding through contact tracing.

|  | No. of suspected cases | No. of suspected cases tested positive | No. of contacts of suspected cases | No. of contacts tested positive |
| --- | --- | --- | --- | --- |
| Residents | 27 | 7 (25.9%) | 440 | 39 (8.9%) |
| Staff  members | 59 | 12 (20.3%) | 605 | 15 (2.4%) |
| Total | 86 | 19 (22.0%) | 1.045 | 54 (5.2%) |

**S5 Underlying medical conditions**

From 96.3% of the 243 infected residents and from 96.7% of the 152 infected staff members information was obtained regarding underlying medical conditions. Residents often had several underlying medical conditions (up to 10). The most commonly reported diseases among residents with COVID-19 were heart diseases, neurological or neuromuscular diseases and psychological diseases. Among the staff members, the most commonly noted diseases were endocrine disorders and lung diseases (see Table 5).

Table S5. Underlying diseases of SARS-CoV-2 positive residents (n = 234) and staff members (n = 147).

| Diseases | No. of residents | No. of staff members |
| --- | --- | --- |
| Heart disease | 103 (44.0%) | 2 (1.4%) |
| Diabetes | 53 (22.6%) | 8 (5.3%) |
| Liver or gall bladder disease | 25 (10.7%) | 0 (0.0%) |
| Neurological or neuromuscular disease | 117 (50.0%) | 5 (3.3%) |
| Suppressed immune system | 4 (1.7%) | 3 (2.0%) |
| Cancer | 31 (13.2%) | 2 (1.4%) |
| Kidney disease | 54 (23.1%) | 4 (2.7%) |
| Lung disease | 30 (12.8%) | 13 (8.8%) |
| Musculoskeletal disorder | 94 (40.1%) | 7 (4.8%) |
| Endocrine disorder | 49 (20.9%) | 17 (11.6%) |
| Gastrointestinal disorder | 42 (17.9%) | 4 (2.7%) |
| Psychological disorder | 127 (54.2%) | 2 (1.4%) |
| Rheumatism | 11 (4.7%) | 1 (0.7%) |
| Urogenital disease | 41 (17.5%) | 0 (0.0%) |

**S6 Hospitalizations**

Out of the group that tested positive for SARS-CoV-2, 32 individuals (27 residents, 5 staff members) were hospitalized (see Table 6). Two of the hospitalized residents were hospitalized twice. The median age of the 32 hospitalized cases was 83 years (ranging from 53 to 96 years). 53% of the hospitalized patients were male. The median duration of stay was 9 days (ranging from less than 24 hours to 28 days), calculated from a total of 27 hospital stays for which the PHDR received both a starting and an end date.

Detailed information about hospital stay was reported to the PHDR for 18 of the 31 hospitalized patients. Out of these 18 patients, 13 (72%) were admitted with symptoms of pneumonia, which in 12 cases was attributed to COVID-19. 4 of 18 patients were admitted for acute renal failure, 3 of whom also had symptoms of pneumonia. Other reasons for admission included heart conditions, embolic events, gastrointestinal complaints, cancer and trauma, with some patients being admitted for multiple reasons.

Two patients received treatment in the intensive care unit for 3 and 4 days, respectively. One of these two patients had to be ventilated, while further 10 hospitalized patients, all suffering from pneumonia, received oxygen during their stay in the hospital.

The most common pre-existing conditions among the 29 hospitalized patients, whose pre-existing conditions were reported were cardiovascular diseases (25, out of which 12 were heart disease), psychiatric disorders (13), renal diseases (10), neurological/neuromuscular diseases (13) and musculoskeletal disorders (8). 4 patients suffered from chronic lung diseases.

**S7 Deaths**

By May 23^rd^, 2020, 50 COVID-19 related deaths (out of a total of 83 such deaths in Reutlingen County) had been reported from nursing homes (see Table 6). The median age of the deceased was 90 years for women (78-106 years; n = 29), and 86 years for men (79-97 years; n = 21). Two nursing homes reported over ten COVID-19 deaths each among their residents (21% of 81 and 20% of 54 COVID-19 patients, respectively).

Four nursing home residents died during their stay in hospital, five within 2-5 days after their discharge from the hospital. Deaths occurred about 10 days after diagnosis (median). The most common underlying medical conditions among the deceased were cardiovascular diseases (36), neurological/neuromuscular diseases (22), psychiatric disorders (22), musculoskeletal disorders (13) and renal diseases (12); four patients had suffered from chronic lung diseases.

Table S6. Number of COVID-19 cases among staff members and residents, number of hospitalizations and deaths among COID-19 cases.

| Nursing home | No. of residents | No. of staff | No. of tested residents | No. of  tested staff | No. of  residents tested positive | No. of staff tested positive | No. of people tested positive total | No. of hospitali-zations | No. of COVID-19 related deaths |
| --- | --- | --- | --- | --- | --- | --- | --- | --- | --- |
| ALT10 | 79 | 117 | 78 | 117 | 56 | 25 | 81 | 3 | 17 |
| ALT7 | 48 | 57 | 47 | 57 | 36 | 18 | 54 | 7 | 11 |
| ALT11 | 116 | 156 | 116 | 150 | 34 | 26 | 60 | 5 | 8 |
| ALT30 | 46 | 47 | 46 | 47 | 34 | 20 | 54 | 5 | 3 |
| ALT2 | 80 | 100 | 80 | 95 | 32 | 13 | 45 | 3 | 7 |
| ALT4 | 33 | 61 | 32 | 53 | 17 | 12 | 29 | 2 | 0 |
| ALT26 | 56 | 60 | 55 | 55 | 3 | 6 | 9 | 1 | 0 |
| ALT27 | 50 | 54 | 47 | 47 | 2 | 4 | 6 | 0 | 0 |
| ALT25 | 49 | 57 | 47 | 48 | 12 | 3 | 15 | 1 | 0 |
| ALT36 | 46 | 44 | 44 | 43 | 2 | 2 | 4 | 0 | 0 |
| ALT19 | 70 | 95 | 70 | 78 | 3 | 1 | 4 | 1 | 2 |
| ALT20 | 67 | 86 | 66 | 82 | 1 | 6 | 7 | 1 | 0 |
| ALT13 | 50 | 65 | 47 | 65 | 1 | 1 | 2 | 1 | 1 |
| ALT17 | 86 | 130 | 86 | 123 | 1 | 0 | 1 | 0 | 0 |
| ALT14 | 50 | 64 | 41 | 55 | 1 | 0 | 1 | 1 | 0 |
| ALT8 | 70 | 85 | 69 | 84 | 6 | 6 | 12 | 0 | 1 |
| ALT18 | 42 | 53 | 39 | 51 | 1 | 1 | 2 | 1 | 0 |
| ALT21 | 36 | 35 | 35 | 34 | 0 | 2 | 2 | 0 | 0 |
| ALT28 | 92 | 162 | 88 | 126 | 0 | 1 | 1 | 0 | 0 |
| ALT33 | 52 | 53 | 52 | 48 | 0 | 1 | 1 | 0 | 0 |
| ALT3 | 56 | 61 | 56 | 55 | 0 | 1 | 1 | 0 | 0 |
| ALT16 | 33 | 41 | 31 | 34 | 0 | 1 | 1 | 0 | 0 |
| ALT6 | 41 | 63 | 40 | 61 | 0 | 1 | 1 | 0 | 0 |
| ALT23 | 47 | 63 | 47 | 63 | 0 | 1 | 1 | 0 | 0 |
| ALT9 | 62 | 57 | 56 | 47 | 0 | 0 | 0 | 0 | 0 |
| ALT22 | 2 | 1 | 2 | 0 | 0 | 0 | 0 | 0 | 0 |
| ALT31 | 40 | 79 | 39 | 78 | 0 | 0 | 0 | 0 | 0 |
| ALT5 | 19 | 21 | 18 | 15 | 0 | 0 | 0 | 0 | 0 |
| ALT32 | 44 | 62 | 40 | 51 | 0 | 0 | 0 | 0 | 0 |
| ALT24 | 65 | 80 | 61 | 75 | 0 | 0 | 0 | 0 | 0 |
| ALT15 | 81 | 78 | 79 | 65 | 0 | 0 | 0 | 0 | 0 |
| ALT37 | 29 | 35 | 29 | 24 | 0 | 0 | 0 | 0 | 0 |
| ALT1 | 47 | 55 | 47 | 51 | 0 | 0 | 0 | 0 | 0 |
| ALT29 | 62 | 74 | 60 | 67 | 0 | 0 | 0 | 0 | 0 |
| ALT12 | 47 | 61 | 47 | 58 | 0 | 0 | 0 | 0 | 0 |
| ALT35 | 35 | 27 | 35 | 26 | 0 | 0 | 0 | 0 | 0 |
| ALT34 | 73 | 78 | 70 | 73 | 1 | 0 | 1 | 0 | 0 |

**S8 Number of examined and infected staff members and nursing home residents in Phases 1-3**

| Table S7. Number of examined and infected staff members and nursing home residents in phases 1-3 | | | | | | | | | | | | | | | |
| --- | --- | --- | --- | --- | --- | --- | --- | --- | --- | --- | --- | --- | --- | --- | --- |
|  | **Phase 1 (March 8 - April 5, 2020)** | | | | | **Phase 2 (April 5 - 20, 2020)** | | | | | **Phase 3 (April 21 - May 25, 2020)** | | | | |
| **Nursing home** | **No. of tested**  **residents** | **No. of residents tested positive** | **No. of staff**  **tested** | **No. of staff tested positive** | **Total no. of people tested positive** | **No. of tested**  **residents** | **No. of residents tested positive** | **No. of staff tested** | **No. of staff tested positive** | **Total no. of people tested positive** | **No. of tested**  **residents** | **No. of residents tested positive** | **No. of staff**  **tested** | **No. of staff tested positive** | **Total no. of people tested positive** |
| ALT30 | 15 | 12 | 16 | 6 | 18 | 31 | 19 | 30 | 10 | 29 | 15 | 4 | 26 | 4 | 8 |
| ALT11 | 12 | 5 | 19 | 4 | 9 | 104 | 23 | 123 | 14 | 37 | 83 | 6 | 137 | 9 | 15 |
| ALT4 | 1 | 1 | 7 | 4 | 5 | 31 | 16 | 31 | 7 | 23 | 0 | 0 | 18 | 2 | 2 |
| ALT20 | 4 | 0 | 10 | 5 | 5 | 61 | 1 | 68 | 0 | 1 | 62 | 0 | 55 | 1 | 1 |
| ALT26 | 4 | 0 | 7 | 4 | 4 | 54 | 4 | 48 | 2 | 6 | 2 | 0 | 3 | 0 | 0 |
| ALT19 | 3 | 2 | 14 | 1 | 3 | 65 | 1 | 63 | 0 | 1 | 9 | 0 | 6 | 0 | 0 |
| ALT8 | 14 | 2 | 15 | 1 | 3 | 57 | 0 | 66 | 3 | 3 | 65 | 4 | 75 | 2 | 6 |
| ALT7 | 2 | 2 | 1 | 0 | 2 | 45 | 30 | 51 | 16 | 46 | 13 | 4 | 37 | 2 | 6 |
| ALT2 | 2 | 2 | 0 | 0 | 2 | 70 | 18 | 87 | 12 | 30 | 60 | 12 | 74 | 2 | 14 |
| ALT10 | 1 | 1 | 1 | 1 | 2 | 75 | 50 | 105 | 20 | 70 | 26 | 6 | 92 | 4 | 10 |
| ALT18 | 1 | 1 | 0 | 0 | 1 | 38 | 0 | 51 | 1 | 1 | 1 | 0 | 0 | 0 | 0 |
| ALT27 | 0 | 0 | 2 | 1 | 1 | 47 | 2 | 46 | 3 | 5 | 1 | 0 | 3 | 0 | 0 |
| ALT9 | 0 | 0 | 0 | 0 | 0 | 56 | 0 | 46 | 0 | 0 | 0 | 0 | 1 | 0 | 0 |
| ALT33 | 3 | 0 | 5 | 0 | 0 | 50 | 0 | 43 | 1 | 1 | 4 | 0 | 5 | 0 | 0 |
| ALT22 | 0 | 0 | 0 | 0 | 0 | 2 | 0 | 0 | 0 | 0 | 0 | 0 | 0 | 0 | 0 |
| ALT31 | 1 | 0 | 3 | 0 | 0 | 35 | 0 | 77 | 0 | 0 | 3 | 0 | 2 | 0 | 0 |
| ALT5 | 1 | 0 | 0 | 0 | 0 | 17 | 0 | 14 | 0 | 0 | 0 | 0 | 1 | 0 | 0 |
| ALT3 | 2 | 0 | 4 | 0 | 0 | 55 | 0 | 54 | 1 | 1 | 2 | 0 | 1 | 0 | 0 |
| ALT32 | 2 | 0 | 0 | 0 | 0 | 39 | 0 | 47 | 0 | 0 | 0 | 0 | 5 | 0 | 0 |
| ALT16 | 0 | 0 | 0 | 0 | 0 | 31 | 0 | 33 | 1 | 1 | 0 | 0 | 1 | 0 | 0 |
| ALT24 | 0 | 0 | 0 | 0 | 0 | 57 | 0 | 71 | 0 | 0 | 8 | 0 | 6 | 0 | 0 |
| ALT17 | 0 | 0 | 2 | 0 | 0 | 84 | 1 | 122 | 0 | 1 | 3 | 0 | 2 | 0 | 0 |
| ALT25 | 0 | 0 | 2 | 0 | 0 | 43 | 2 | 43 | 1 | 3 | 29 | 10 | 34 | 2 | 12 |
| ALT28 | 0 | 0 | 1 | 0 | 0 | 82 | 0 | 115 | 0 | 0 | 2 | 0 | 13 | 1 | 1 |
| ALT13 | 0 | 0 | 0 | 0 | 0 | 47 | 1 | 65 | 1 | 2 | 1 | 1 | 1 | 0 | 1 |
| ALT14 | 0 | 0 | 1 | 0 | 0 | 41 | 1 | 55 | 0 | 1 | 0 | 0 | 2 | 0 | 0 |
| ALT15 | 0 | 0 | 0 | 0 | 0 | 79 | 0 | 65 | 0 | 0 | 1 | 0 | 1 | 0 | 0 |
| ALT37 | 0 | 0 | 0 | 0 | 0 | 29 | 0 | 24 | 0 | 0 | 0 | 0 | 1 | 0 | 0 |
| ALT1 | 0 | 0 | 0 | 0 | 0 | 47 | 0 | 50 | 0 | 0 | 0 | 0 | 3 | 0 | 0 |
| ALT29 | 0 | 0 | 1 | 0 | 0 | 57 | 0 | 59 | 0 | 0 | 54 | 0 | 55 | 0 | 0 |
| ALT6 | 1 | 0 | 1 | 0 | 0 | 37 | 0 | 60 | 1 | 1 | 5 | 0 | 1 | 0 | 0 |
| ALT12 | 0 | 0 | 0 | 0 | 0 | 47 | 0 | 58 | 0 | 0 | 2 | 0 | 0 | 0 | 0 |
| ALT23 | 1 | 0 | 0 | 0 | 0 | 47 | 0 | 63 | 1 | 1 | 1 | 0 | 2 | 0 | 0 |
| ALT35 | 0 | 0 | 1 | 0 | 0 | 32 | 0 | 25 | 0 | 0 | 5 | 0 | 1 | 0 | 0 |
| ALT36 | 0 | 0 | 0 | 0 | 0 | 44 | 2 | 42 | 2 | 4 | 0 | 0 | 2 | 0 | 0 |
| ALT21 | 0 | 0 | 2 | 0 | 0 | 35 | 0 | 32 | 1 | 1 | 3 | 0 | 1 | 1 | 1 |
| ALT34 | 3 | 0 | 2 | 0 | 0 | 66 | 0 | 72 | 0 | 0 | 4 | 1 | 17 | 0 | 1 |
